# Trinidad and Tobago: A decade of dementia research

**DOI:** 10.1590/S1980-57642014DN84000005

**Published:** 2014

**Authors:** Nelleen Baboolal, Gershwin Davis, Amanda McRae

**Affiliations:** 1 Department of Clinical Medicine Sciences; 2 Department of Paraclinical Sciences; 3 Department of Preclinical Sciences. Faculty of Medical Sciences, University of the West Indies, St Augustine

**Keywords:** caregiver burden, biomarkers, dementia prevalence socioeconomic impact

## Abstract

In 2003, academic staff members at The University of the West Indies Faculty of Medical Sciences St Augustine Trinidad and Tobago combined their expertise to make strides in Alzheimer's and Dementia research in Trinidad and Tobago. Dr. Nelleen Baboolal, Dr. Gershwin Davis and Professor Amanda McRae began developing a project that has produced significant results by examining not only the epidemiology of dementia, but the associated risk factors; caregiver burden and ultimately establishing biomarkers for the disease. This review is an account of our results from a decade of dementia research and how they are contributing toward mitigating the dementia tsunami in Trinidad and Tobago.

## Abstract (pt)

Em 2003, os membros da equipe acadêmica da Faculdade de Ciências Médicas St Augustine, da Universidade de West Indies, Trinidad e Tobago, combinaram seus conhecimentos para promover avanços nas pesquisas sobre doença de Alzheimer e demência em Trinidad e Tobago. Dr. Nelleen Baboolal, Dr. Gershwin Davis e Professora Amanda McRae começaram a desenvolver um projeto que tem produzido resultados significativos através da análise não só da epidemiologia da demência, mas também dos fatores de risco associados, sobrecarga do cuidador e, ultimamente, estabelecendo biomarcadores para a doença. Esta avaliação representa um relato de nossos resultados de uma década de pesquisa demência e como eles estão contribuindo para mitigar o tsunami de demência em Trinidad e Tobago.

## INTRODUCTION

The Republic of Trinidad and Tobago is a twin island state located at the southernmost tip of the Caribbean archipelago. Its multi-ethnic population of approximately 1.3 million consists of East Indian 40.3%; African 39.6%; mixed 18.4%; and 1.7% belong to other ethnic groups.^1^ Ethnic differences have been observed in many important complex chronic illnesses and acute infections. There is an established predominance of early-onset diabetes mellitus in the South Asian population and a higher prevalence and severity of hypertension in African citizens.^2^ There is an increase in metabolic syndrome across all ages of the population. Furthermore and highly significant, is that Trinidad and Tobago is an ageing population. At present, the elderly population of Trinidad and Tobago stands at 13 percent or 177, 676 persons over the age of 60 years.^1^ This figure is expected to increase to 17.7% by 2025. In essence, this population resides amid an arsenal of risk factors currently considered to potentiate cognitive decline. As the population of the Trinidad and Tobago ages there is a need to prepare the policy makers for what could be considered a cognitive deficit tsunami. One can ask: are we ready for the social and physical changes that accompany aging?

For many, dementia remains a stigma. Consequently, the elderly may have a tendency not to report early symptoms of memory loss. This can even extend to family members who may be reluctant to accept or report that an elderly relative is displaying cognitive decline. As the population ages it is essential to prepare the population for the road ahead.

There have been some improvements in this direction with the establishment of eight senior activity centers and organizations such as the Senior Achievers, Golden Years and the Trinidad and Tobago Association for Retired Persons and the Alzheimer's Association of Trinidad and Tobago. These social organizations are essential for mental stimulation and to decrease loneliness.

Epidemiological data is fundamental to optimize planning of services and policy.^3,4^ The absence of functional and reliable data collection is obscuring the reality of dementia in this region. There has been a gross underestimation of the number of cases of dementia in the country, as many patients with dementia live at home they are not being accounted for.

Furthermore, there is growing concern about caregivers. Many caregivers have no knowledge about the clinical features of the disorder. Therefore they are ill-prepared to cope with the behavioral changes which accompany the progression of dementia. This group needs to be educated about the frustration and exhaustion encountered by caring for an elderly relative with dementia. Many caregivers are elderly themselves.

Delaying the onset of Alzheimer's disease is a high priority for any given population. Identifying strategies to slow down the progression of AD as well as other aged-related cognitive disorders has become a collective world-wide mission. Progress has been made to decipher areas which have the potential to lower the risk of AD. Simple interventions including diet, education, exercise, and increasing cognitive activity are among several factors recently considered ways of reducing the risk of Alzheimer's disease. Thus, it is important for a given population to identify risk factors which may be pertinent to their region and environment, thereby allowing interventions to slow down the onset of dementia.

Trinidadians are at great risk for dementia. Firstly, there is a high prevalence of diseases which are risk factors for dementia.^2^ Secondly, the World Health Organization has estimated that the prevalence of Dementia in the Caribbean and Latin America is the highest in the world.^5^

What is being done in Trinidad and Tobago to mitigate the dementia tsunami? To this end, the present review will provide an account of the efforts of three academic staff members at the University of the West Indies at the Faculty of Medical Sciences to help prepare the population and policy makers for this tsunami.

## RESEARCH TEAM

One could consider that this is a result of being at the right place at the right time. The University of the West Indies promotes research and specifically encourages faculty members to form research clusters. This was the case for Dr. Nelleen Baboolal, Senior Lecturer in Psychiatry, Dr. Gershwin Davis, Senior Lecturer in Chemical Pathology, and Professor Amanda McRae, Professor of Human Anatomy who had individually conducted dementia research. We met and decided to pool our expertise in order to make strides in Alzheimer's and Dementia research in Trinidad and Tobago.

For the last decade we have consistently held research meetings on Wednesdays. Our dedication and commitment to dementia research has indeed been very rewarding. We began developing a four-stage project that could yield breakthrough results by examining not only the epidemiology of Dementia, but the associated risk factors; caregiver burden and ultimately establishing biomarkers for the disease. This is a collaborative effort not on a specific project but on the theme of Alzheimer disease and Dementia and Mild Cognitive Impairment (MCI). To that end, we established and incorporated Dementia Awareness and Research of Trinidad and Tobago (DARTT) which is a voluntary non-profit organization which aims to educate the population, promote brain health, diagnose afflicted persons, support patients, families and caregivers, conduct research on Alzheimer's disease and establish the prevalence of dementia and its economic burden.

In 2003, a very successful memory clinic was established at a tertiary teaching hospital. This provided the infrastructure for both clinical and basic research. Patients were seen and clinical evaluations, blood and neuro-radio imaging investigations and assessment of caregivers were done.

Another successful approach from our collaboration has been outreach. We have all participated in several local TV programs aimed at promoting dementia research and extending awareness of dementia to the general population.

Realizing that several of our research projects would need age-matched control subjects we made contact with organizations such as the Senior Achievers and the Golden Years. Both of these organizations are composed of dynamic seniors aged 60 and above. We were invited to their monthly meetings to address issues related to dementia and its awareness. They all agreed to take the Mini-Mental State Exam (MMSE)^6^ and donate blood samples. We arranged a day at each organization and administered the MMSE. Blood samples were taken on a different day. Another organization where we have had a conspicuous presence is the Alzheimer's Association of Trinidad and Tobago (AATT) where we interacted with a monthly support group for caregivers. In addition to caregivers at Dr. Baboolal's memory clinic, we have also administered caregiver questionnaires to those attending the AATT. Furthermore, we have played active roles in events organized by the AATT during Alzheimer's awareness month in September.

In 2005, we also launched a MPhil/PhD Neuroscience degree at the University of the West Indies, the first such degree program in the English-speaking Caribbean. Our first PhD Neuroscientist graduated in 2014.

Additionally, between 2011 and 2013 the research of two graduate students, who completed the Masters in Public Heath at the University of the West Indies, was supervised by Dr. Baboolal. Their research projects examined "The Plight of the Caregiver: A Study of the Burden Placed upon Caregivers of Patients with Dementia in Trinidad" and "The Prevalence of depression among the Elderly who attend Senior Activity Centres in Trinidad".

From the very outset of our collaboration, Dr. Baboolal considered that one of the most important achievements from our group would be to establish the prevalence of dementia and its economic impact in Trinidad and Tobago. Determining a central figure such as the prevalence of dementia in Trinidad and Tobago is critical because for the first time, it will provide the Government, and anyone else who wants to know, with a picture of how common dementia is and the socio-economic cost that comes with it. Results will also have enormous transformative implications for policy, as they will highlight the economic reasons for investing in dementia. Though we realized how essential it was to establish the nationwide prevalence of dementia and its economic impact in Trinidad and Tobago, organizing this project, including funding, took several years and in fact the project actually began in 2012. While we were preparing for the nationwide prevalence study, we conducted a prevalence of dementia investigation in persons attending senior activity centers, those in nursing homes, and persons collecting welfare checks.

Professor. R. Stewart of the Institute of Psychiatry, King's College, London has been instrumental in guiding us with the preparations of the prevalence of dementia study. He has conducted several workshops and will analyze the data.

In view of the high prevalence of diabetics in Trinidad and Tobago,^2^ we also investigated cognitive function in type 2 diabetic patients. This collaboration benefited from the expertise of Professor S. Teelucksingh of The University of the West Indies.

Our collaborative efforts have generated several publications, book chapters, workshops and conference presentations at international conferences, including the International Conference on Alzheimer Disease, Vas Cog, American Association of Clinical Chemistry Conference and Caribbean Health Research Conference.

Our major research accomplishments include:

Biomarkers, cognitive assessment in diabetic patients, caregiver burden, prevalence of dementia in three different settings, and the nationwide prevalence and economic impact of dementia in Trinidad and Tobago. The sections below provide a synopsis of results obtained from some of these various research themes.

## BIOMARKERS

One key facet of the project of our group is developing a serum screening biomarker for the disease that could introduce a paradigm shift in the way we approach the healthcare maintenance of the elderly. This is due to the fact that a serum marker would provide a universal means to differentiate Alzheimer's Disease (AD) from other dementias, as well as establish early detection of the disorder.

The Trinidadian population may have a raised risk for dementia because hypertension, diabetes and cerebrovascular disease are common.^2^

Based on the high prevalence of the above disorders we chose to investigate factors which could predict or assist in discriminating types of dementias in our population from healthy seniors.^7^

We selected the amino acid homocysteine (tHcy), C-reactive protein (CRP) and serum sialic. Elevated circulating levels of homocysteine are an independent risk factor for stroke.^8^ Furthermore, elevated levels of tHcy have been linked to cognitive decline.^8,9^ CRP is considered to have a link to cardiovascular disorders and has been investigated in relationship to the development of certain dementias.^10^ Serum sialic acid is a potent cardiovascular and renal risk factor as it is increased in cerebrovascular disease and in patients with micro- and macro-vascular complications of diabetes.^11^ In view of the relationship of sialic acid to disorders considered risk factors for dementia, it may also be a predictor of cognitive decline.

The investigation included 51 healthy elderly individuals who were members of a seniors group plus 27 persons with dementias of the Alzheimer's type (AD), persons with Alzheimer's disease or persons with pure vascular dementia (VaD). The MMSE was administered and all patients were subjected to interview, physical examination and neurological examination. The clinical/biochemical characteristics of both groups were compared.

Plasma tHcy was determined on the Abbot AxSym using FPIA. Serum CRP concentrations were measured using the Tina-Quant sCRP (Latex) high sensitive immunoturbidimetric assay on the Roche/ Hitachi 912 Automatic Analyzer. Serum sialic acid was measured by spectrophotometric assay using standard chemicals and reagents. For the dementia patients, the main clinical diagnoses were AD, 18 (67%) and VaD, 9 (33%).^7^

When the controls were compared with all patients as a group, the MMSE and sialic acid differed significantly, with MMSE scores being higher and sialic acid levels lower.in controls. Patients with AD had significant differences in the MMSE scores and sialic acid scores, but not for tHcy and CRP values when compared with controls. In patients with VaD however, significant differences were obtained for both MMSE scores and tHcy but not for sialic acid or CRP.

Several research studies have shown that the concentration of sialic acid in serum is elevated in pathological states when there is damage to tissue, tissue proliferation and inflammation The latter has in recent times reemerged as an important aspect of the pathogenesis of Alzheimer disease.^12^ Our findings suggest that elevations in serum sialic acid levels could be related to AD pathology. In this regard, it is of interest that a recent study^13^ has demonstrated that reduction in sialic acid protects PC 12 cells from B amyloid toxicity. From a speculative point of view, the elevated levels of sialic acid may reflect an increase in the deposition of B amyloid. Further studies are necessary to elucidate the relationship between elevated sialic acid levels and ongoing AD pathology.

The finding that sialic acid levels were significantly higher in patients with AD compared to controls and not different with respect to VaD is unlike the results for tHcy, where notable differences were found between VaD and controls. This suggests that there may be different mechanisms at work in the pathogenesis of the two conditions.^7^

We have also identified another biomarker which appears to have diagnostic potential. Previous studies have demonstrated that the cerebrospinal fluid (CSF) from AD patients contains an antibody directed against microglia (MgAbs).^14^ The rapidly expanding field of neuroinflammation has revealed that immunocompetent microglia play an early role in the events leading to AD pathology.^15^ It should be remembered that a biomarker is a substance such as an antibody or protein, which is usually present in either the cerebrospinal fluid or blood. According to the criteria of the Consensus Report of the Working Group on Molecular and Biochemical Markers of AD, an ideal biomarker should: be able to detect a fundamental feature of AD neuropathology; and be validated in neuropathologically-confirmed AD cases; be precise (able to detect AD early in its course and distinguish it from other dementias); reliable; non-invasive; simple to perform; and inexpensive.^16^ Thus, it appears reasonable to propose that MgAbs could be a potential biomarker for AD.

This antibody has shown in both clinically^14^ and neuropathologically confirmed AD cases^17^ to be present at a greater frequency in the CSF compared to other dementias. Among patients in Trinidad and Tobago, we further demonstrated that serum MgAbs can distinguish AD from healthy age-matched controls.^18^ There was no significant difference between the presence of MgAb in VaD patients compared to controls.^18^

For us, an exciting milestone was reached in 2008, after conducting a workshop entitled "Biomarkers for Dementia. Is there a role?" at the American Association of Clinical Chemistry Conference in Washington DC. This attracted the attention of a major UK-based diagnostic company. Subsequently, collaboration developed between this company and the UWI to further the development of MgAbs as a diagnostic biomarker for Alzheimer's disease.

**Figure 1:**
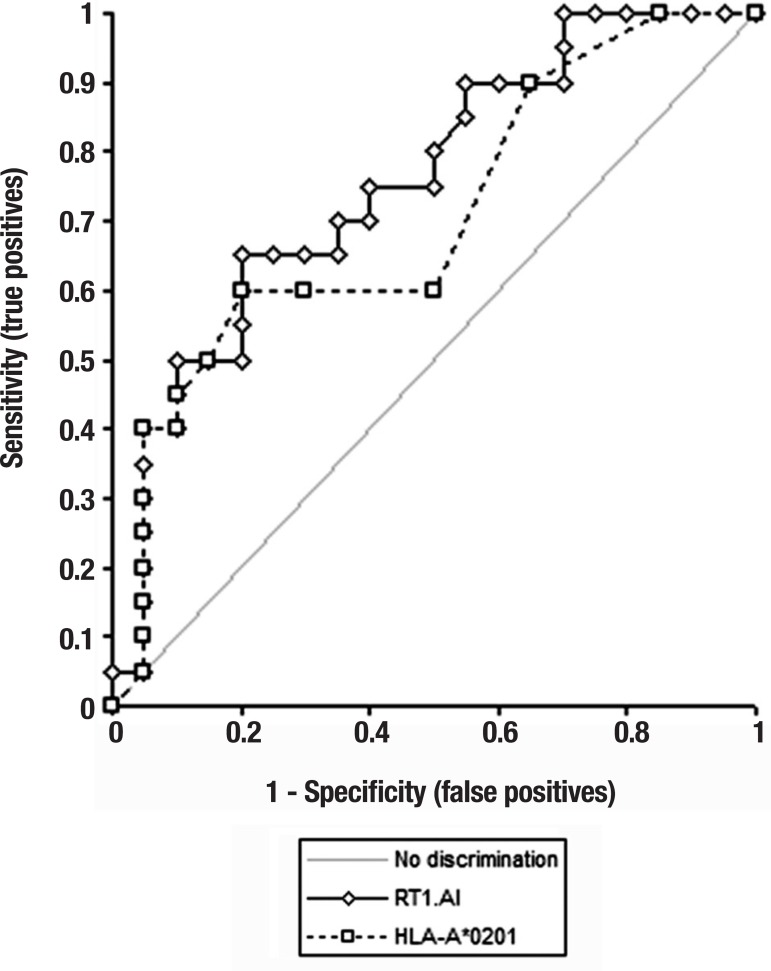
ROC curves using RT1.A^1^ and HLA-A^*^0201 as the antigen.

This collaboration was pursued. We identified major histocompatibility complex 1 (MHCI) as the microglial surface antigen to which autoantibodies are directed in AD patients. ELISAs were established using two distinct forms of MHCI as the antigen. One form was HLA.A*0201, the most commonly expressed form of MHCI in humans, whilst rat RT1.A^l^ was also used to provide a more direct comparison with the rat brain cross-sections previously employed for immunocytochemistry. Data was analyzed by constructing receiver operator characteristic (ROC) curves. When the cerebrospinal fluid (CSF) samples from the cohort of 20 patients with Alzheimer's disease and the 20 individuals without Alzheimer's disease were tested on the newly developed ELISA platform there was a clear and statistically significant association between the presence of anti-MHC1 antibodies and the presence of Alzheimer's disease. Using RT1.A as the antigen in the ELISA, the area under the curve (AUC) was 0.756 (p=0.0004) and when using HLA-A*0201 as the antigen the AUC was 0.705 (p=0.0071). The ROC curve analysis suggested that for the CSF we have a new ELISA test that is at best good at distinguishing AD patients from controls (Figure 1). This test should be studied not only in patients with Alzheimer's disease but also in other groups of patients such as those classified as MCI. Further work is in progress to establish an ELISA test for serum MgAbs.

As shown when the data was analyzed by constructing receiver operator characteristic (ROC) curves, using RT1.A^1^ as the antigen in the ELISA, the area under the curve (AUC) was 0.756 (p=0.0004) and when using HLA-A^*^0201 as the antigen AUC was 0.705 (p=0.0071). The (ROC) shows the effectiveness of the new ELISA test in discriminating between true cases of disease from non-disease. The maximum value for AUC 1 indicates a perfect test and a value of 0.5 - the straight line through zero - indicates no discriminative value.

## COGNITIVE TESTING IN DIABETES MELLITUS PATIENTS

The elevated prevalence of diabetes mellitus in Trinidadian citizens is an indication that a large part of the population is at risk of developing dementia. It has been established that patients with diabetes have increased deposition and decreased clearance of amyloid,^19^ as well as increased incidence of hypoglycemia and hyperglycemia which contributes to cognitive impairment. Patients with a diagnosis of diabetes have nearly double the risk of developing both dementia and mild cognitive impairment compared to non-diabetics in the elderly population.^20^ In view of the link between diabetes mellitus and cognitive decline, we considered that it would be relevant to investigate cognitive function in patients with diabetes mellitus in Trinidad and Tobago. There were 96 patients with type 2 diabetes mellitus and 87 age-matched non-diabetic controls in this study. Demographic data was obtained from both diabetic patients and healthy age-matched controls.

In order to determine normal cognitive function, MCI, or dementia the following tests: Addenbrooke`s Cognitive Examination Revised (ACE-R), MMSE (Mini-Mental State Exam), Color trails-1, Color trails-2, Picture-Number Matching, Word Recall and Digit Span Forward and Backward were administered to both patients and controls.

Of all these tests, only performance on the ACE-R - a brief cognitive screening instrument sensitive to early stages of dementia- was significantly different when comparing persons with diabetes to controls.

These findings suggest that the ACE-R could be a useful screening test in primary care for detecting the presence of early cognitive dysfunction in diabetics. The MMSE was not sensitive enough to pick up mild cognitive impairment in Diabetic patients.

## CAREGIVING IN TRINIDAD AND TOBAGO

Dementia and cognitive impairment are the leading chronic disease contributors to disability, and particularly dependence, among older people worldwide. The need for support from a caregiver often starts early in the dementia journey, intensifies as the illness progresses over time, and continues until death.^21^ Caregivers include family, friends, as well as community and paid caregivers who may or may not be family. The World Alzheimer Report 2013 reveals the global Alzheimer's epidemic is creating a shortage of caregivers and lack of support for family members.

Unpaid care for persons with dementia is provided by family, friends and community, and care is generally referred to as 'informal' care. Paid care is referred to as 'formal' care. Family caregivers and paid caregivers share much in common. They all carry out difficult, demanding and socially useful roles, with minimal training and preparation. In Trinidad and Tobago, a significant number of persons suffering from dementia are cared for at home by an informal caregiver. Caring for a person with AD and other dementias is associated with significant risk to the caregiver's health and well-being.^22^

The term "caregiver burden" is used to describe the physical, emotional and financial cost of providing care.^23^ The Zarit Burden Interview (ZBI) is a standardized, validated, reliable tool for assessment of the burden of caregivers for dementia patients.

There has been no assessment of caregiver burden in Trinidad and Tobago. Thus, we administered the ZBI to caregivers to evaluate the degree of burden in caregivers of dementia patients in a Trinidadian population. The ZBI was developed by Zarit and co-workers in 1985^24^ and comprises a 22-item questionnaire with a five-item response set ranging from "never" to "nearly always" graded on a scale from 0 to 4, according to the presence or intensity of an affirmative response. Based on the total score, individuals were classified as having little or no burden (0-20), mild to moderate burden (21-40), moderate to severe burden (41 to 60), or severe burden (61-88). The questions refer to the caregiver/patient relationship and evaluate the caregiver's health status, psychological well-being, finances, and social life. The caregiver burden is evaluated by means of the total score obtained from the sum total of questions. We also included an evaluation of the possible risk factors associated with higher burden. The effects of different factors including patient and caregiver age, gender, years of education, relationship between the patient and caregiver as well as the patient's symptom duration and degree of cognitive impairment were investigated.

Informed consent was obtained from all caregivers and informed consent by proxy was obtained for the investigation of patient characteristics. Seventy-five patients diagnosed with dementia along with their caregivers were included in the study. Patients were diagnosed using the DSM IV TR,^25^ the MMSE,^6^ clinical evaluations, laboratory investigations and brain CT or MRI. Demographic characteristics of patients and their caregivers were recorded

The GHQ-28 was administered to all caregivers. It contains 28 items that, through factor analysis, have been divided into four sub-scales. The GHQ-28 is the most well-known and popular version of the GHQ. It is used to detect psychiatric disorder in the general population and within community or non-psychiatric clinical settings such as primary care or general medical out-patients. It assesses the respondent's current state and asks if that differs from his or her usual state. It is therefore sensitive to short-term psychiatric disorders but not to long-standing attributes of the respondent.

Data revealed that there were 56 female (74.7%) and 19 male (25.3%) patients with 36 (48%) more than 79 years of age. Patient ages ranged from 59 to 94 years, mean 77.6 years, S.D. 8.3. Thirty-six (48%) patients were of African descent, 13 (17.3%) of East Indian descent and 26 (34.6%) other ethnicities. Thirty (40%) were married, 32 (42.7%) widowed, 6 (8%) were single and 5 (6.7%) divorced. Duration of symptoms of dementia was 0.08 to 14 years, mean 4.3 years, S.D. 3.5.

Most caregivers were females 61 (81.3%) and 48 (64%). Caregiver ages ranged from 27 to 86 years, mean 57.3, s.d. 15.2. The majority of the caregivers were offspring 32 (42.7%) and spouses 18 (24%). Forty-one (54.7%) caregivers were married and 35 (46.7%) had secondary school education.

The ZBI scores ranged from 0 to 63 with a mean of 22.7, S.D.14.7. and 41 (55%) overall had some degree of burden. According to the Zarit Buren Interview scores, 45% experienced little or no burden, 43% experienced mild to moderate burden, 11% moderate to severe burden and 1% severe burden. The GHQ Scores were >47 in 49.3% of caregivers (p=0.01).

The preponderance of females with dementia in this study is significant and in keeping with international studies.^26-28^ Persons who were not of East Indian or African descent were described as belonging to a minority ethnic group. It is therefore surprising that in our study there were only 13 (17.3%) persons of East Indian descent with dementia since, according to the 2011 Census, persons of East Indian descent comprised 35.4%, African descent 34.2%, mixed persons comprised 22.8%, and other ethnic groups 1.4%.^2^ Whether this is due to lower numbers of East Indian patients with dementia is a finding that requires exploration in future studies.

Caregiver burden was significantly associated with the patient being male (p=0.03) and the patient belonging to a minority ethnic group (p=0.07). Our study answers the question 'who are the caregivers?'. Akin to other reports^29,30^ we too have found that over 80% of caregivers are women, more specifically middle-aged women. It is notable that the majority of these women are adult children and spouses. Though we have found that the degree of burden with respect to the Zarit Burden Interview score did not differ from other caregivers, this is a group that is at increased risk for stress-related medical conditions since they form the majority of the caregivers. Although other studies report higher caregiver burden in female caregivers,^31^ there was no correlation between gender of the caregiver and burden in the present study.

Our study found no significant correlation between relationship of the caregiver to the patient, cohabiting status of the caregiver, marital status of the caregiver, educational level of the caregiver and occupation of the caregiver.

The GHQ Scores, a measure of psychiatric morbidity, were >47 in 49.3% of caregivers. Higher caregiver burden scores using the ZBI were associated with higher caregiver GHQ scores, a finding that supports previous studies.^32^ Research has shown that caregivers of demented patients are nearly twice as likely to have symptoms of depression compared with caregivers of non-demented people. As such, caregivers should be advised to protect their personal time, watch out for symptoms of depression such as crying more, sleeping/eating more or less than usual and lack of interest in usual activities.

Our study found that 45% of the caregivers experienced little or no burden. This might reflect the easy acceptance of dementia for the elderly in the Trinidadian population where taking part of the elderly is a normal intergenerational experience. In Trinidad, it is not culturally usual to institutionalize aged family members (demented or otherwise) and the elderly are commonly a part of normal living in many households. More than half of the caregivers (55%) had moderate to severe burden

Another issue that this study hints at is the impact of this unpaid care giving on the financial health of these individuals who, in there middle age, have other responsibilities including taking care of their own families. This is an area that would need follow-up studies. This is especially important since the average duration of dementia in our study was 4.4 years.

In the absence of state and private agencies to support the elderly, the familial care networks have been and continue to be the main source of support for seniors in Trinidad and Tobago.^33^ Our findings underscore the global impact of caring for a person with dementia and support the need for caregiver support, education, training and access to medical care.

## THE PREVALENCE AND ECONOMIC COST OF DEMENTIA IN TRINIDAD AND TOBAGO

In 2012, we launched the Prevalence of Dementia and its Socio-economic Burden study in Trinidad and Tobago.

The project is a collaboration between the Dementia Awareness Research of Trinidad & Tobago (DARTT), Faculty of Medical Science and the Centre for Health Economics, Faculty of Social Sciences, The University of the West Indies.

The fundamental purpose of this project is to determine the prevalence of dementia in persons aged 60 and above in all municipalities in Trinidad and Tobago. This study will also determine the associated cost and implications for the family and caregivers, health care system and economy of Trinidad and Tobago.

The prevalence study will use validated 10/66 interview protocols^34,35^ together with a socioeconomic questionnaire generated by the Center of Health Economics unit of The University of the West Indies.

The protocols of the key research instrument, the 10/66, have successfully established the prevalence of dementia in a number of countries including Cuba, Mexico, Peru, Venezuela, the Dominican Republic, India and China.^35,36^ With the use of an extended 10/66 protocol and collaboration with Professor Robert Stewart (Institute of Psychiatry, King's College London) a founding member of the 10/66 research group, it is certain that our result can be compared with data from other countries. This would have a significant effect on the interpretation of results as to future trends and impact of dementia in our local setting.

Dementia will be diagnosed using an abbreviated and recently validated version of the 10/66 assessment schedule.^35^ The component measures will consist of the Community Screening Instrument for Dementia (CSI-D), the CERAD 10-word list recall task, and the EURO-D depression scale.

Standard practice in 10/66 surveys to date has been to recruit 2000 participants aged 65 years and over per site.^34,35^

Our survey in Trinidad and Tobago will improve on the usual 10/66 design in two ways:

1. It will be the first 10/66-style survey of a national population (rather than a geographic catchment);
2. It will be the first such survey to adopt age-stratified sampling. The latter approach is feasible in Trinidad and Tobago because of the recent national census which provides the opportunity to sample within age ranges (something not possible for most countries) and is particularly valuable for a disorder such as dementia whose prevalence increases exponentially with age (approximately doubling with every 5-year increase in age after 65).

The proposed sample will be recruited in the following strata: 500 participants aged 60-69 years, 500 aged 70-79, 500 aged 80-89, 500 aged 90+, randomly sampled throughout all municipalities in Trinidad and Tobago.

To ensure that our door-to-door 10/66 surveys are conducted in a similar manner as those performed in the other countries, Professor Robert Stewart, our International consultant, conducted a training workshop for the 30 selected field workers.

The field work has now been completed and the data is being analyzed so that determination of the prevalence of dementia and its socioeconomic burden in Trinidad is imminent.

The impacts of this study are as follows:

1. Firstly, this study is the first of its kind in Trinidad and Tobago and will allow our policymakers to comprehend both the current prevalence and impact of dementia.
2. Impact of the socioeconomic findings from our study. It is anticipated that the findings of this study will build awareness of the full cost of dementia (including some cost elements that may not have been as obvious to those not directly impacted by dementia). This study will detail the needs of the individual and households affected by dementia with a view to enhancing the welfare and wellbeing of such individuals and households
3. To raise public awareness about dementia which in turn should: reduce stigmas surrounding the disorder, encourage early diagnosis, help family and caregivers cope with the disorder, lead to the adoption of healthier life styles which could postpone the onset of dementia.
4. One of the anticipated impacts of our study is that policy makers will make dementia a national priority by adopting and implementing a National Dementia Plan.

**Conclusion**. The research presented in the this review is the result of the efforts of three staff members of the Faculty of Medical Sciences at the University of the West Indies who pooled their expertise to advance knowledge about dementia. It has been indeed a rewarding journey and the fruits of our research are beginning to be revealed. For one, we are also very pleased to have been asked to be members of a committee that will produce the National Dementia plan for Trinidad and Tobago. Secondly, we consider that through our various types of outreach we have been increasing dementia awareness in Trinidad and Tobago.

It is our goal, as we continue with our research, that it makes a difference for those affected by the disorder either directly or indirectly, that it allows policy makers to give a high priority to dementia research, and that systems will be put into place to decrease caregiver burden. All of these should indeed mitigate the dementia tsunami in Trinidad and Tobago.
